# Two Novel QTLs for the Harvest Index that Contribute to High-Yield Production in Rice (*Oryza sativa* L.)

**DOI:** 10.1186/s12284-021-00456-1

**Published:** 2021-02-10

**Authors:** Hiroki Saito, Yoshimichi Fukuta, Mitsuhiro Obara, Asami Tomita, Tsutomu Ishimaru, Kazuhiro Sasaki, Daisuke Fujita, Nobuya Kobayashi

**Affiliations:** 1 Tropical Agriculture Research Front (TARF), Japan International Research Center for Agricultural Sciences (JIRCAS), Okinawa, 907-0002 Japan; 2 Japan International Research Center for Agricultural Sciences (JIRCAS), Ibaraki, 305-8686 Japan; 3 Present address: Faculty of Agriculture, Okayama University, Okayama, 700-8530 Japan; 4 Hokuriku Research Station, Central Region Agricultural Research Center (CARC), National Agriculture and Food Research Organization (NARO), Niigata, 943-0193 Japan; 5 Present address: Faculty of Agriculture, Saga University, Saga, 840-8502 Japan; 6 Present address: Institute of Crop Science, National Agriculture and Food Research Organization (NARO), Tsukuba, 305-8518 Japan

**Keywords:** Rice (*Oryza sativa* L.), Harvest index, Source ability, Yield potential, QTL

## Abstract

**Background:**

The harvest index (HI) is a measure of the biological success of forming harvestable products. However, our understanding of the genetic basis of HI in rice (*Oryza sativa* L.) is limited, because it is a complex trait consisting of various yield-related traits and physiological attributes. YTH183 is a high-yielding line with large panicles and high HI derived from a cross between the Indica Group variety IR 64 and the NPT line IR 69093-41-2-3-2 (YP5).

**Results:**

Here, we detected two novel QTLs for HI, designated *qHI5.1* on chromosome 5 and *qHI8.1* on chromosome 8, by using 155 recombinant inbred lines (RILs) derived from the cross between IR 64 and YTH183. The YTH183 allele at *qHI5.1* contributed to a wide grain, resulting in heavy grain weight and panicle weight, and was consistently effective under the different environmental conditions of subtropical (Ishigaki) and temperate (Tsukuba) regions. Genetic polymorphism revealed that *qHI5.1* was identical to *GSE5/GW5*, which is known to control the grain weight. On the other hand, although *qHI8.1* functioned additively with *qHI5.1* for higher HI, it did not show any significant effect on grain or panicle weight. In addition, its effects on HI were shown only in the first seasons at Ishigaki but not at Tsukuba or in the second season at Ishigaki.

**Conclusion:**

Our results indicate that *qHI5.1* controls the grain size, regardless of whether environmental conditions are of subtropical or temperate regions, while *qHI8.1* might be involved in controlling the physiological processes of source ability or the translocation of photosynthesis products from vegetative organs to grains depending on environmental conditions during the maturing stage. These QTLs will be useful genetic resources for future breeding programs to break through the ceiling of maximum yield in Indica Group varieties.

**Supplementary Information:**

The online version contains supplementary material available at 10.1186/s12284-021-00456-1.

## Background

Rice (*Oryza sativa* L.) is one of the most important cereal crops in the world. More than half of the world’s population depends on rice as their staple food, and, in Africa, the overall demand for rice has outstripped local production (Zenna et al. 2017). Moreover, ongoing global warming exposes rice production to serious risks, such as extreme heavy rain, flooding, and disastrous droughts. To address the demand for food for over nine billion people by 2050, and, further, to respond to the challenges posed by global warming, the genetic improvement of yield potential in rice-breeding programs is one of the most important strategies.

Since the release in 1966 of IR 8, the first semi-dwarf variety with high yield potential, the International Rice Research Institute (IRRI) has aimed to further improve the yield potential of IR-series varieties and has proposed new-plant-type varieties (NPTs) based on the “plant type (ideotype) concept” (Donald 1968; Yoshida 1972; Peng et al. 1994, 2008; Khush 1995). NPTs are varieties harboring low tiller number, few unproductive tillers, and a large number of grains per panicle, and a phenotype expected to result in high yield potential (Peng et al. 1994). NPTs developed from the crosses between the tropical Japonica Group varieties and the improved Indica Group varieties have been released since 1993; however, grain yields have been disappointing because of low biomass production and poor grain filling (Peng et al. 2008). To achieve high grain filling and increase yield potential, particularly in terms of improvement of the sink-source balance, it is necessary to simultaneously improve both sink capacity (a large number of spikelets per panicle, heavy grains, and stable grain filling) and source ability (carbohydrate supply) to accommodate the large sink size under high temperatures.

An informative indicator of the sink-source balance is the harvest index (HI). HI is defined as the ratio of harvestable parts to biomass and is taken as a measure of biological success in partitioning photosynthates to the harvestable product (Donald and Hamblin 1976; Sinclair 1998). Recent breeding programs have achieved great increases in yield by improving HI. Hay (1995) concluded that yield increase in rice was a consequence of the incorporation of dwarfing genes that increased the harvest index by around 0.3 to 0.5. Therefore, HI is a useful indicator to consider in the breeding of new high-yielding varieties.

Molecular genetic research has detected several QTLs for HI in rice (Hittalmani et al. 2003; Zhang et al. 2004, 2017; Marri et al. 2005; Sabouri et al. 2009; Li et al. 2012). HI is a quantitative trait controlled by multiple genes (QTLs) for numbers of panicles, grain weight, grain numbers, plant biomass, and photosynthetic carbohydrate accumulation and translocation, and the expressions of these QTLs for HI are affected by the environment (Hittalmani et al. 2003; Li et al. 2012; Zhang et al. 2017). However, there are few studies on the beneficial genetic basis of HI independent of environmental factors.

A total of 334 introgression lines with a genetic background of IR 64 were developed with NPTs under the IRRI-Japan Collaborative Research Project (Fujita et al. 2009). Among them, YTH183 exhibited higher yield and HI than those of IR 64 under various environmental conditions, such as water-saving (aerobic), tropical wet and dry, irrigated lowland and upland, and temperate climate conditions (Kato et al. 2011; Uddin et al. 2016; Ishimaru et al. 2017; Takai et al. 2019). It has been demonstrated that the higher yield potential and greater dehydration avoidance in YTH183 might be conferred, at least in part, by introgressed segments on chromosomes 5 and 6, respectively (Kato et al. 2011). Fujita et al. (2010) and Ishimaru et al. (2017) reported that one QTL for grain weight was mapped on chromosome 5. Thus, genetic analysis using YTH183 is expected to reveal another genetic factor for the sink-source balance or the source ability in addition to grain weight (i.e., sink capacity).

In this study, we attempted to identify QTLs for HI by using recombinant inbred lines (RILs) derived from crosses between IR 64 and YTH183. To confirm the regional effects of detected QTLs on yield-related traits and physiological attributes, a cross-locational investigation was conducted in locations with subtropical and temperate climates in Japan. The present study provides novel information to improve the HI of the Indica Group varieties through better sink-source balance or source ability.

## Materials and Methods

### Plant Materials and Cultivation

An Indica Group variety IR 64 and YTH183 (IR84636-13-12-2-6-3-3-2-2-B), harboring chromosome segments from the Tropical Group varieties in Indonesia (Fujita et al. 2009), were used as parental lines to develop recombinant inbred lines (RILs) for QTL analysis. YTH183 is one of the introgression lines derived from the cross between IR 64 and an NPT accession (IR69093-41-2-3-2, designated as YP5) (Fujita et al. 2010), and therefore has a limited number of genomic regions introduced into IR 64 (Obara et al. 2014). A total of 155 RILs and their parents were grown in paddy fields at JIRCAS in Ishigaki, Okinawa, Japan (24.38°N,124.19°E) and in Tsukuba, Ibaraki, Japan (36.05°N,140.08°E) with the cropping schedule described in Table 1. Ishigaki and Tsukuba are classified as having subtropical and temperate climates, respectively. Note that two cropping seasons per year are possible in Ishigaki. A total of four trials—three years (2016, 2017, and 2018) in the first cropping at Ishigaki and a single year (2017) at Tsukuba—were performed to identify QTLs for HI.

**Table 1:** Sowing date, transplanting date and investigation traits of each cropping season

Site	Ishigaki				Tsukuba
Latitude / Longitude	N: 24.38^o^ / E: 124.19^o^				N: 36.05^o^ / E: 140.08^o^
Year	2016	2017	2018		2017
Season	First	First	First	Second	–
Abbeviation of crop season	2016ISG_F	2017ISG_F	2018ISG_F	2018ISG_S	2017TKB
Sowing	February 16, 2016	February 14, 2017	February 14, 2018	July 13, 2018	April 21, 2017
Transplanting	March 15, 2016	March 14, 2017	March 14, 2018	August 1, 2018	May 21, 2017
Details of plant materials and experimental methods	155 RILs and their parents for QTL analysis	155 RILs and their parents for QTL analysis Two parents for HI transition by 2-day intervals after heading	155 RILs and their parents for QTL analysis	24 selected RILs for two-way ANOVA test between two QTLs for HI	155 RILs and their parents for QTL analysis
Traits for investigation^a^	PW	PW	PW	PW	PW
	CW	CW	CW	CW	CW
	TW	TW	TW	TW	TW
			PN		PN
		PW (2-day intervals)	TS		
		CW (2-day intervals)	GN		
		TW (2-day intervals)	FR		
			GWt		
			GL		
			GWd		

^a^ These abbreviation means as follows: *PW* Panicle weight, *CW* Culm and leaf weight, *TW* Total weight, *PN* Panicle number per plant, *TS* Total number of spiklets per panicle, *GN* Number of filled grains per panicle, *FR* Fertility ratio, *GWt* Grain weight, *GL* Grain length, *GWd* Grain width

Additionally, to confirm the effects of the detected QTLs on HI in the different cropping season at Ishigaki, six lines each of four different genotype combinations at two QTLs for HI were selected from those RILs, and we cultivated at Ishigaki in the second cropping season of 2018. Twenty 28-day-old seedlings per line were transplanted, one per hill, 18 cm apart, in two rows 36 cm apart. An organic slow-release fertilizer (5.2-5.2-5.2 g/m^2^ N-P-K at Ishigaki and 4.8-6.4-3.2 g/m^2^ N-P-K at Tsukuba) was applied as a basal fertilizer.

### Evaluation of Yield-Related Traits

We investigated three yield-related traits in all cropping seasons at Ishigaki and Tsukuba—total weight per plant (TW), culm weight per plant (CW), and panicle weight per plant (PW)—which were used to calculate the ratio of panicle weight to total weight (PW/TW = harvest index [HI]) (Table 1). In the first cropping season of 2018 at Ishigaki, in addition to the above three traits, we investigated other traits: panicle number per plant (PN), total number of spikelets per panicle (TS), number of filled grains per panicle (GN), ratio of filled grains per panicle (GN/TS = fertility ratio [FR]), and 1000-grain weight (GWt). Further, we also investigated grain length (GL) and grain width (GWd) of approximately 50 grains per plant each using the software SmartGrain (Tanabata et al. 2012) in the first cropping season of 2018 at Ishigaki. In the 2017 cropping season at Tsukuba, we investigated PW, CW, TW, and PN.

In the first cropping season of 2017 at Ishigaki, we also investigated the developmental transition of the ratio of panicle weight to total weight in two parental lines at two-day intervals from 4 weeks before heading until harvest. Six undamaged plants from each line were investigated in all trials, and the average value was taken to be representative for each line. Whole plants at 35 days after heading were harvested and dried in a well-ventilated storage house for measurement.

### QTL Analysis

Genomic DNA was extracted from frozen leaves of individual plants from the RILs by the method described in Monna et al. 2002 with slight modifications. A total of 22 SSR markers and 74 KASP markers (McCouch et al. 2010; LGC Genomics, Teddington, UK), which were located on the chromosome segment from YP5, were used for genotyping of the 155 RILs (Table 2, Supplemental Fig. 3).

**Table 2:** Number of KASP and SSR markers on twelve chromosomes

Chromosome	1	2	3	4	5	6	7	8	9	10	11	12	Total
Number of markers	8	1	0	0	36	9	1	11	9	18	1	2	96
Average recombinat value (cM)	1.3	0.0	0.0	0.0	3.7	2.1	0.0	2.1	14.8	1.8	0.0	0.4	3.6

QTL analyses for yield-related traits were performed using the R/qtl software package (Broman et al. 2003). Interval mapping (IM) was performed for each trait using the “scanone” function with default settings to calculate the LOD value for each marker. The LOD threshold was obtained based on the permutation test (1000 permutations, *P* = 0.05) for each trait. The additive effect was calculated as (the mean value of the AA genotype) – (the mean value of the AA genotype + the mean value of the BB genotype) / 2. The phenotypic variance explained (PVE) by each QTL was estimated by 1–10^−2LOD/n^, where *n* indicates the sample size and LOD indicates the LOD value from “scanone.”

## Results

### HI and Yield-Related Traits in the Two Parental Lines

**Fig. 1:**
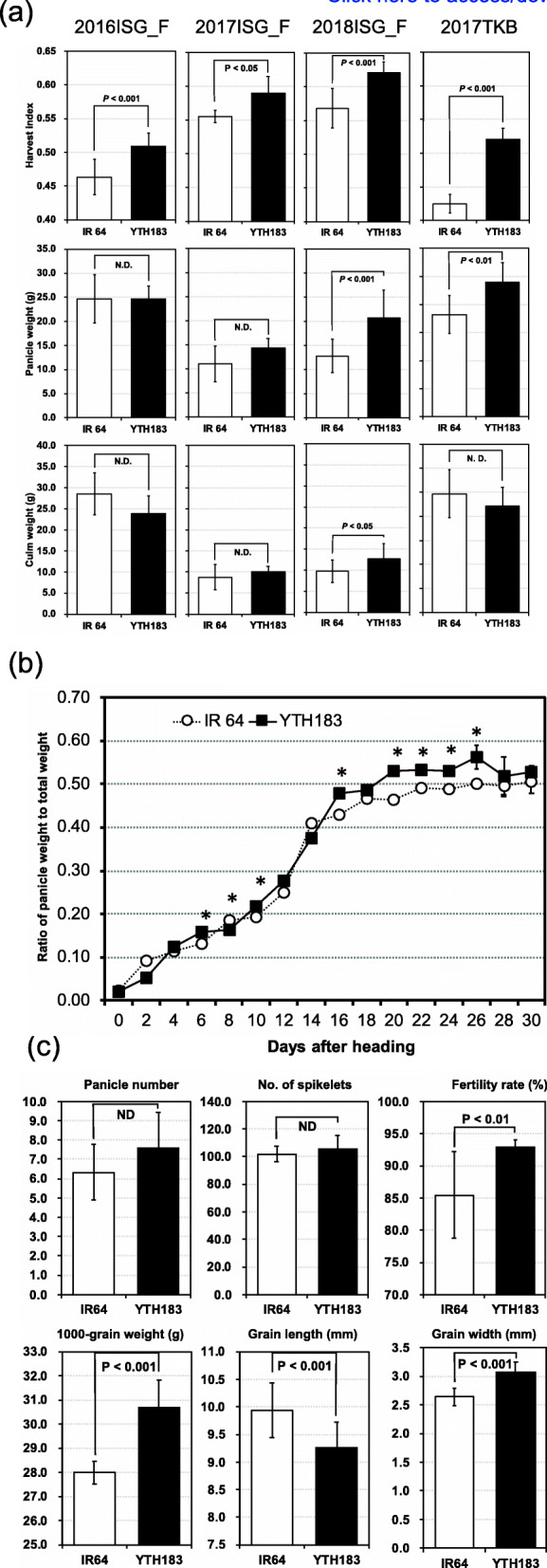
Comparison of HI and yield-related traits between IR 64 and YTH183. Data are expressed as means ± SD (*n* = 6 plants). **a** HI, panicle weight, and culm (and leaf) weight in Tsukuba (2017TKB) and three first-cropping seasons at Ishigaki in 2016, 2017, and 2018 (2016ISG_F, 2017ISG_F, and 2018ISG_F). The *P* value indicates statistical analysis by Student’s t-test. N.D. indicates no significant difference. **b** Transitions of HI between IR 64 and YTH183 in the first cropping at Ishigaki in 2017. The asterisks represent a significant difference between the two parents (*P* < 0.05) based on Student’s t-test. **c** Comparison of six yield-related traits between IR 64 and YTH183 in the first cropping at Ishigaki in 2018. The *P* value indicates statistical analysis by Student’s t-test. N.D. indicates no significant difference

The heading dates of IR 64 at Ishigaki were on May 30 in 2016, June 3 in 2017, and May 30 in 2018; on the other hand, the heading dates of YTH183 at Ishigaki were on May 28 in 2016, June 2 in 2017, and May 28 in 2018 (Supplemental Table 1). For 3 years from 2016 to 2018, HIs of YTH183 were significantly higher than those of IR 64 both at Ishigaki and at Tsukuba in 2017 (Fig. 1a). PWs of YTH183 were similar or higher than those of IR 64, and CWs varied from lower to higher than those of IR 64 among four trials. This means that PW is a more stable trait than CW. The dry matter productions of PW and CW at Ishigaki in 2017 and 2018 were lower than in the other two trials at Ishigaki in 2016 and at Tsukuba in 2017, and the HIs at Ishigaki in 2017 and 2018 were also higher than these. These results indicate that HIs became bigger under the condition of low dry matter production compared with high. The condition of the fertilizer (4.8 kg/10a, nitrogen basis: N) applied for cultivations was the same in these four investigations, but the basic soil fertility conditions between Ishigaki and Tsukuba were different, and the fields were also different among the three trials at Ishigaki. Basically, the variations in dry matter productions of IR 64 and TH183 might be influenced by the soil fertility conditions. Comparing the developmental transitions in the ratio of panicle weight to total weight between YTH183 and IR 64, remarkably significant differences were found in the late maturing stage (Fig. 1b). In the yield-related traits, although there was no significant difference in PN or TS between YTH183 and IR 64, GWt and FR were higher in YTH183 than in IR 64, resulting in a higher PW in YTH183 (Fig. 1c). Furthermore, GWd was higher in YTH183 than in IR 64, while GL was lower (Fig. 1c). It is therefore considered that the large grain size in YTH183 contributes to the higher HI and yield than that of IR 64.

### QTL Analyses

**Fig. 2:**
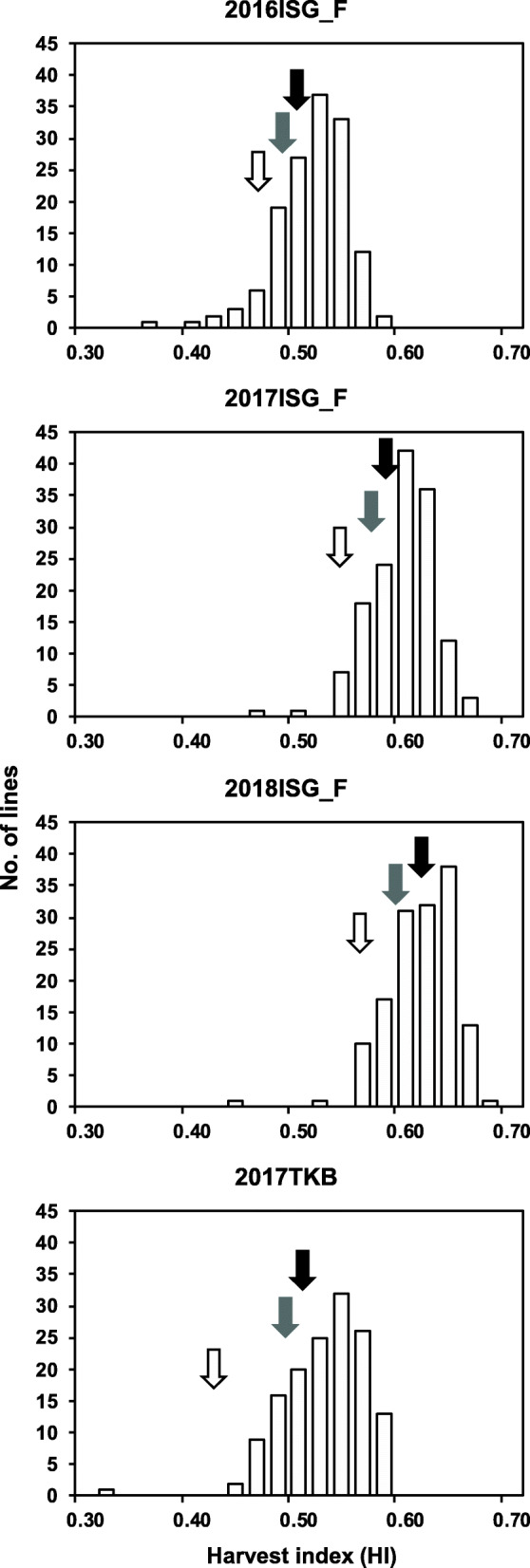
Frequency distributions of HIs of RILs in the cropping seasons at Ishigaki (ISG) and Tsukuba (TKB). The white, black, and gray arrows indicate, respectively, the HI of IR 64, YTH183, and the average of the RILs

The HIs of RILs in the 3 years were continuously distributed with transgressive segregates (Fig. 2, Supplemental Table 2). In addition, significant positive correlations were observed between each cropping season (Table 3). Compared with the correlations among the three cropping seasons at Ishigaki, the correlations between the cropping season at Tsukuba and the cropping seasons at Ishigaki were low (Table 3).

**Table 3:** The correlations of HI in RILs among four cropping seasons

Season	2016ISG_F	2017ISG_F	2018ISG_F	2017TKB
2016ISG_F	–			
2017ISG_F	0.723 ***	–		
2018ISG_F	0.681 ***	0.796 ***	–	
2017TKB	0.680 ***	0.486 ***	0.548 ***	–

*** represents a significant correlation between the two cropping seasons at *P* < 0.001 level

**Fig. 3:**
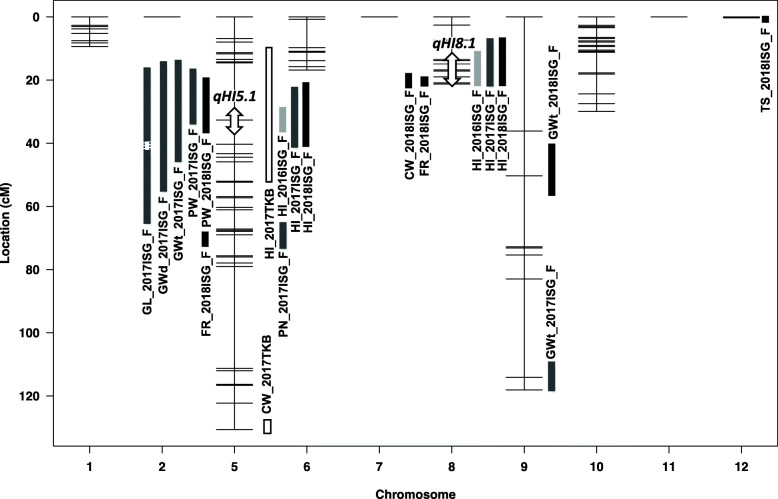
Linkage map developed by the genetic analysis of RILs and QTLs identified in this study. The vertical bars indicate the range in which the LOD value of each QTL exceeds the threshold. The double-headed arrows on chromosomes 5 and 8 indicate the putative region where the responsible genes for HI would be located. Abbreviations are as follows: Panicle weight (PW), culm and leaf weight (CW), grain weight (GWt), grain length (GL), and grain width (GWd)

**Fig. 4:**
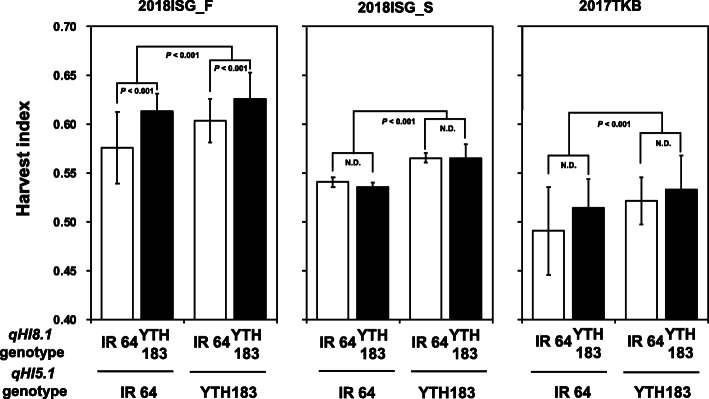
The mean of harvest index in 2018ISG_F, 2018ISG_S, and 2017TKB for different genotype classes between *qHI5.1* and *qHI8.1* in RIL populations. Genotypes are represented by the nearest marker loci, RM1089 (*qHI5.1*) and 8,099,836 (*qHI8.1*). The *P* value indicates the statistical analysis by Student’s t-test. N.D. indicates no significant difference

QTL analyses identified a total of twenty QTLs for eight agricultural traits, including HI, on chromosomes 5, 8, 9, and 12 (Fig. 3, Table 4). Among them, QTLs for HI were identified in the regions on chromosomes 5 and 8 for the 3 years at Ishigaki. At Tsukuba, only the QTL on the region of chromosome 5 was identified, and that on chromosome 8 was not (Fig. 3, Table 4, Supplemental Fig. 1). We designated the two QTLs on chromosomes 5 and 8 as *qHI5.1* and *qHI8.1*, respectively. Both of the YTH183 alleles at these QTLs additively contributed to the high HI without any genetic interactions in the 3 years of cropping at Ishigaki (Fig. 4).

**Table 4:** QTLs identified in this study

Trait	Chr. ^a^	2016ISG_F					2017ISG_F					2018ISG_F					2017TKB				
		LOD	Peak position (cM)	QTL range (cM)	Additive effect^b^	PVE (%) ^c^	LOD	Peak position (cM)	QTL range (cM)	Additive effect^b^	PVE (%) ^c^	LOD	Peak position (cM)	QTL range (cM)	Additive effect^b^	PVE (%) ^c^	LOD	Peak position (cM)	QTL range (cM)	Additive effect^b^	PVE (%) ^c^
Harvest index (HI)	5	2.42	36.9	36.2–37.5	0.009	6.9	5.48	32.8	22.0–41.4	0.013	15.0	6.62	34.4	20.2–44.1	0.015	17.9	4.67	50.7	13.7–57.1	0.015	13.0
	8	4.87	20.9	10.4–21.3	0.013	13.5	4.55	13.8	7.6–21.3	0.012	12.7	3.57	13.8	8.5–21.23	0.011	10.1					
Panicle weight (PW)	5						3.19	23.90	15.6–32.4	0.515	9.0	2.82	25.2	19.4–31.5	0.775	8.0					
Culm and leaf weight (CW)	5																3.10	130.7	125.6–130.7	−1.461	8.8
	8											3.10	20.4	11.0–21.3	−0.626	8.8					
Total number of spikelets per panicle (TS)	12											2.50	2.50	0–0.4	−3.000	7.2					
Fertility rate (FR)	5											2.80	2.80	66.2–74.3	1.214	8.0					
	8											3.17	20.89	20.0–21.3	1.239	9.0					
Grain weight (GWt)	5											14.78	34.7	8.1–50.8	1.248	35.5					
Grain length (GL)	5											9.04	32.7	20.2–42.0	−0.130	23.6					
	8											2.44	19.0	18.9–19.1	− 0.085	7.0					
	9											2.74	50.4	47.7–53.1	−0.068	7.8					
Grain width (GWd)	5											36.7	34.1	0–75.9	0.147	66.3					
	9											3.8	117.7	105.6–118.2	0.062	10.7					

^a^ “Chr.” indicates “chromosome”

^b^ A positive value for the additive effect indicates that the YTH183 allele increases the trait

^c^ “PVE” indicates a percent of the variation explained

In the region where *qHI5.1* is located, a QTL for PW was detected (by analyses in 2017 and 2018). Further, QTLs for GWt, GL, and GWd were also located in the same region. In this region, the *GSE5/GW5* gene for grain weight is reported (Duan et al. 2017; Liu et al. 2017). Therefore, we investigated genomic polymorphism of the *GSE5* gene between YTH183 and IR 64, and found that YTH183 harbored an insertion at the promoter region of this gene (Supplemental Fig. 2).

On the other hand, in the region where *qHI8.1* is located, QTLs for CW, FR, and GL were detected (by analysis in 2018). However, we did not detect any QTLs, including for HI, at this region in the cropping at Tsukuba (Fig. 3, Table 4). Interestingly, although *qHI5.1* showed a significant effect on the HI in the second cropping season at Ishigaki in 2018, *qHI8.1* did not (Fig. 4). During the maturing stage in the second cropping season at Ishigaki in 2018, the average daily temperature was as low as it was in the cropping season at Tsukuba. The average temperatures during the maturing stage at Tsukuba and the second cropping at Ishigaki were remarkably lower than those of the three first cropping seasons at Ishigaki (Supplemental Table 3). This difference in temperatures during the maturing stage might have influenced the effect of *qHI8.1*.

## Discussion

The harvest index (HI) is used to measure biological success in the formation of harvestable products. However, the genetic basis of HI in rice is not well understood because it is a complex trait comprising a number of yield-related traits and physiological attributes. Under the IRRI-Japan Collaborative Research Project, 334 introgression lines with the genetic background of IR 64 were developed to enhance yield potential by backcrossing between nine NPT varieties and one Japanese high-yielding cultivar as the donor parents and IR 64 as the recurrent parent (Fujita et al. 2009). Among those introgression lines, YTH183 showed remarkably higher yield and greater adaptability to both tropical and temperate regions than IR 64 (Takai et al. 2019). In this study, we genetically analyzed the yield potential of YTH183 from the perspective of HI. Our results showed that high correlations were found among the 3 years of HI at Ishigaki, whereas low correlations were found between the 3 years of HI at Ishigaki and the HI at Tsukuba (Table 3), suggesting that RILs showed different performances between Ishigaki and Tsukuba. HI is a complex trait and susceptible to environmental conditions, and it is difficult to find a genetic factor. Nonetheless, our results clarified the fact that two major QTLs for HI (*qHI5.1* and *qHI8.1* detected on chromosomes 5 and 8, respectively) contributed to the yield potential of YTH183. It is noteworthy that *qHI5.1* was consistently detected under the different climatic conditions of Ishigaki (subtropical) and Tsukuba (temperate) (Fig. 3, Table 4), suggesting that *qHI5.1* may be a useful genetic factor for the genetic improvement of HI in Indica Group varieties grown in both temperate and subtropical regions. In the same region on chromosome 5, QTLs for panicle weight (PW), grain weight (GWt), and grain width (GWd) were also identified, indicating that this QTL increased the total sink size through increasing grain size. This QTL did not have any negative effect on yield-related traits, such as culm weight (CW) or fertility rate (FR). Our results are consistent with those of previous research showing that the improved HI of YTH183 is attributable to the enlarged sink capacity due to large grain size (Kato et al. 2011). Further, previous studies have shown that an introgressed segment in YTH183 contains a major QTL for grain size (*qSW5*) on chromosome 5 (Shomura et al. 2008), and using the 334 introgression lines identified a QTL for grain weight (*qGW5*) in the same region as *qSW5* (Fujita et al. 2009). Recently, Duan et al. (2017) and Liu et al. (2017) identified a previously unrecognized gene (*GSE5/GW5*) at the *qSW5/qGW5* locus. They showed that natural variations in the promoter of *GSE5/GW5* contribute to grain size diversity. In this study, we found a DNA polymorphism between IR 64 and YTH183 (an insertion in YTH183) in the promoter region of the *GSE5/GW5* gene (Supplemental Fig. 2). The sib lines derived from the same parent (YP5) as YTH183 with the insertion significantly showed heavier grain weight than those without the insertion (Supplemental Fig. 4). We therefore consider that *qHI5.1* is identical to *GSE5/GW5.*

Shomura et al. (2008) compared the rice varieties of the Japonica and Indica Groups, and they then identified a major QTL for grain size as *qSW5*, which is identical to *GSE5/GW5*, and the Japonica allele clearly contributed heavier grain weight. YTH183 was developed by the backcross breeding between a new plant type variety, IR69093-41-2-3-2 (YP5) harboring chromosome segments from the Tropical Japonica Group rice varieties and IR 64 as the recurrent parent. We therefore consider that *qHI5.1* is identical to *GSE5/GW5*, which might be widely harbored in Japonica Group varieties from tropical to temperate, and that the allele of YTH183 contributes to the wide grain, resulting in the higher GWt, PW, and HI in YTH183 than in IR 64. Further, because *qHI5.1* was stably identified under different environmental conditions, it is possible that this QTL could become one of the widely useful genetic factors to increase HI without the negative effect of environmental conditions, especially high temperatures during the maturing stage such as in tropical or subtropical regions. It still needs to be considered that *qHI5.1* might be co-located with *GSE5/GW5* in the same chromosome region, and the identity will have to be confirmed based on the gene isolation of *qHI5.1*.

At the *qHI8.1* region, no other QTLs were identified other than a QTL for leaf weight (LW) in 2018. Kato et al. (2011) demonstrated that the source supply for grain growth, i.e., the concentration and amount of non-structural carbohydrate in vegetative organs at anthesis, was largely not improved in YTH183. On the other hand, Ishimaru et al. (2017) suggested that the higher HI in YTH183 is supported by greater photosynthate translocation from sources to sinks during the maturing stage. They showed greater non-structural carbohydrate depletion during the grain-filling stage and a higher number of vascular bundles in the panicle neck of YTH183, suggesting enhanced capacity for assimilate transport to the developing panicles during the maturing stage. Laza et al. (2006) identified QTLs for HI on chromosomes 8 and 11 by using RILs derived from a cross between IR 72 and an NPT variety that was a sibling of the donor variety (YP5) of YTH183. They indicated that these QTLs might be involved in the difference in grain-filling abilities between the Indica and Japonica Groups. Therefore, *qHI8.1* might be involved in the ability of the photosynthate to be translocated from the source to the sink organs. Interestingly, *qHI8.1* did not have a significant effect on HI in the cropping at Tsukuba or in the second cropping at Ishigaki. This result might be explained by differences in the environmental conditions during the maturing stage: The daily average temperature during the maturing stage was higher in the first cropping season at Ishigaki than in the second cropping at Ishigaki and the cropping at Tsukuba (Supplemental Table 3); therefore, the effectiveness of *qHI8.1* might be temperature dependent. Welch et al. (2010) reported that a higher minimum temperature reduced yield by analyzing data from 227 intensively managed irrigated rice farms in six important rice-producing countries. Peng et al. (2004) also reported direct evidence of decreased rice yields from increased nighttime temperature associated with global warming. In the metabolic process of grain filling, Yamakawa et al. (2007) showed that the expression level of genes encoding starch- or carbohydrate-metabolizing enzymes and translocators was diminished to 89% of the control on average by exposure to high temperature, suggesting that ripening under high temperature induced the occurrence of grains with various degrees of chalky appearance and decreased weight. Therefore, *qHI8.1* is expected to enhance source ability or the translocation of photosynthesis products from shoots to grains under conditions of higher temperature during the maturing stage. Introgression of *qHI8.1*, in addition to *qHI5.1*, into Indica Group varieties would contribute to enhancement of HI in tropical regions, and in temperate regions where air temperatures during the maturing stage have been progressively increasing (Iizumi et al. 2011). The effects of the two QTLs, *qHI5.1* and *qHI8.1*, under various environmental conditions need to be confirmed using the isogeneic lines for *qHI5.1* and *qHI8.1* and the accumulated line for *qHI5.1* and *qHI8.1* with an IR 64 genetic background.

Studies on inheritance of the HI trait are limited, because it is a complex trait that incorporates the number of grains, grain weight, and number of panicles as the sink capacity, and also incorporates culm and leaf weight, photosynthetic rate, and translocation of the assimilated carbon as the source activity. In addition, although some of these traits are highly genetically controlled, most are environment sensitive. Consequently, it is difficult to identify the QTLs for HI independent of environmental effects. Multi-location or multi-year trials are essential for identifying effective and stable QTLs. Hittalmani et al. (2003) identified eleven QTLs for HI at nine locations in Asia representing different environments; however, no common QTL was detected in all nine locations. Li et al. (2012) identified genetic markers associated with HI in both temperate (Arkansas) and subtropical (Texas) climates, but detected no associated markers in common. In our study, two stable QTLs (*qHI5.1* and *qHI8.1*) for HI were identified by three multi-year field trials in the first cropping season at Ishigaki. Among them, *qHI5.1* was found to contribute to the grain size independent of the environmental conditions, whereas *qHI8.1* was efficient for maturing under high temperatures. Further, YTH183 shows superior performance when cultivated under water-saving conditions and shows high plasticity in root elongation in response to re-watering after drought (Kato et al. 2011; Kano-Nakata et al. 2013). Furthermore, Obara et al. (2014) identified a QTL on chromosome 6 for root length under N-deficient conditions in YTH183. Thus, YTH183 is remarkable for its excellent response to water- and N-deficient conditions in addition to its enhanced sink capacity owing to its enlarged grain size. Our current findings and previous work suggest that YTH183 will likely be a noteworthy source of breeding material that can be exploited in rice-breeding efforts in the near future for breaking through the ceiling of maximum yield in response to environmental change under global warming.

## Conclusion

Here, we demonstrate that two novel QTLs for HI (*qHI5.1* on chromosome 5 and *qHI8.1* on chromosome 8) contribute to the higher HI in YTH183 than in IR 64. *qHI5.1* is identical to *GSE5/GW5* and controls the grain size, regardless of whether environmental conditions are of subtropical or temperate regions. *qHI8.1* functioned additively with *qHI5.1* for higher HI, but showed significant effects on yield-related traits only in the first cropping season in Ishigaki, where the air temperatures were higher during the maturing stage. Therefore, *qHI8.1* may be associated with regulation of the physiological processes of the source ability or the translocation of photosynthesis products from shoots to grains according to temperature at the maturing stage. These two QTLs are therefore anticipated to be effective genetic resources in breeding programs attempting to surmount the maximum yield in Indica Group varieties.

## Supplementary Information

**Additional file 1: Figure S1.** Details of two major QTLs for HI. (a) *qHI5.1* and (b) *qHI8.1*. The *GSE5/GW5* gene is closely linked with RM1089. The dotted lines on the LOD value curves indicate a threshold calculated by a 1000-times permutation test (the value is 2.41). **Figure S2.** Identification of genomic polymorphism between IR 64 and YTH183. **(a)** Schematic image of LOC_Os05g09520. The white boxes, black triangle, and black bar indicate, respectively, the exon region, insertion position, and 1 Kbp scale bar. The two arrows indicate the primer positions, whose sequences are as follows: Forward primer: TCCATTTTATTGGCATCACTCA; Reverse primer: CCCAAATCCCAGGCTACTGAT (from Duan et al. 2017). **(b)** Electrophoresis image of the amplicons. M: 100 bp ladder. **Figure S3.** Graphical genotypes of YTH183. The open and closed bar ﻿represent genotypes at regions corresponding to the IR64 homozygous segment and the YP5 homozygous segment, respectively. The top numbers indicate the chromosome number. **Figure S4.** 1000-grain weight of IR64, YTH183, and the sib lines derived from the same parent (YP5) as YTH183 (Fujita et al. 2009) at Tsukuba in 2015. The black and white bars indicate the IR64-type and YTH183-type alleles at the *GSE5/GW5* locus, respectively. The *P* value indicates the statistical analysis by Student’s t-test. **Table S1.** Heading date of IR 64 and YTH183. **Table S2.** The harvest index of IR 64, YTH183, and their derived RIL population. **Table S3.** Average daily temperature during the maturing stage.

## Data Availability

The datasets generated during and analyzed during the current study are available from the corresponding author on reasonable request.

## Abbreviations

NTPs: New-plant-type varieties
HI: Harvest index
RILs: Recombinant inbred lines
TW: Total weight per plant
CW: Culm weight per plant
PW: Panicle weight per plant
PN: Panicle number per plant
TS: Total number of spikelets per panicle
GN: Number of filled grains per panicle
FR: Fertility ratio
GWt: 1000-grain weight
GL: Grain length
GWd: Grain width
