# Supplementary material for: Two Novel QTLs for the Harvest Index that Contribute to High-Yield Production in Rice (Oryza sativa L.)
Source: Rice (N Y). 2021 Feb 10;14:18. doi: 10.1186/s12284-021-00456-1 (PMC7876197; doi:10.1186/s12284-021-00456-1)
Supplement: Supplementary file 1 — Additional file 1: Figure S1. Details of two major QTLs for HI. (a) qHI5.1 and (b) qHI8.1. The GSE5/GW5 gene is closely linked with RM1089. The dotted lines on the LOD value curves indicate a threshold calculated by a 1000-times permutation test (the value is 2.41). Figure S2. Identification of genomic polymorphism between IR 64 and YTH183. (a) Schematic image of LOC_Os05g09520. The white boxes, black triangle, and black bar indicate, respectively, the exon region, insertion position, and 1 Kbp scale bar. The two arrows indicate the primer positions, whose sequences are as follows: Forward primer: TCCATTTTATTGGCATCACTCA; Reverse primer: CCCAAATCCCAGGCTACTGAT (from Duan et al. 2017). (b) Electrophoresis image of the amplicons. M: 100 bp ladder. Figure S3. Graphical genotypes of YTH183. The open and closed bar ﻿represent genotypes at regions corresponding to the IR64 homozygous segment and the YP5 homozygous segment, respectively. The top numbers indicate the chromosome number. Figure S4. 1000-grain weight of IR64, YTH183, and the sib lines derived from the same parent (YP5) as YTH183 (Fujita et al. 2009) at Tsukuba in 2015. The black and white bars indicate the IR64-type and YTH183-type alleles at the GSE5/GW5 locus, respectively. The P value indicates the statistical analysis by Student’s t-test. Table S1. Heading date of IR 64 and YTH183. Table S2. The harvest index of IR 64, YTH183, and their derived RIL population. Table S3. Average daily temperature during the maturing stage. [file 12284_2021_456_MOESM1_ESM.docx]

**Supplemental Fig. 1**

Details of two major QTLs for HI. (a) *qHI5.1* and (b) *qHI8.1*. The *GSE5/GW5* gene is closely linked with RM1089. The dotted lines on the LOD value curves indicate a threshold calculated by a 1000-times permutation test (the value is 2.41).

**Supplemental Fig. 2**

Identification of genomic polymorphism between IR 64 and YTH183. **(a)** Schematic image of LOC_Os05g09520. The white boxes, black triangle, and black bar indicate, respectively, the exon region, insertion position, and 1 Kbp scale bar. The two arrows indicate the primer positions, whose sequences are as follows:

Forward primer: TCCATTTTATTGGCATCACTCA;

Reverse primer: CCCAAATCCCAGGCTACTGAT (from Duan et al. 2017). **(b)** Electrophoresis image of the amplicons. M: 100 bp ladder.

**Supplemental Fig. 3**

﻿Graphical genotypes of YTH183. The open and closed bar ﻿represent genotypes at regions corresponding to the IR64 homozygous segment and the YP5 homozygous segment, respectively. The top numbers indicate the chromosome number.

**Supplemental Fig. 4**

1000-grain weight of IR64, YTH183, and the sib lines derived from the same parent (YP5) as YTH183 (Fujita et al. 2009) at Tsukuba in 2015. The black and white bars indicate the IR64-type and YTH183-type alleles at the *GSE5/GW5* locus, respectively. The *P* value indicates the statistical analysis by Student’s t-test.

Supplemental Table 1

| **Supplemental Table 1** |  |  |
| --- | --- | --- |
| Heading date of IR 64 and YTH183 | | |
| Cropping season | IR 64 | YTH183 |
| 2016ISG_F | 5/30 | 5/28 |
| 2017ISG_F | 6/3 | 6/2 |
| 2018ISG_F | 5/30 | 5/27 |
| 2018ISG_S | 10/7 | 10/8 |
| 2017TKB | 8/10 | 8/7 |
|  | | |

Supplemental Table 2

| **Supplemental Table 2** | |  |  |  |  |
| --- | --- | --- | --- | --- | --- |
| The harvest index of IR 64, YTH183, and their derived RIL population. | | | | | |
| **Year** | **Parent** |  |  | **RIL population** |  |
|  | **IR 64 (Mean ± SD)** | **YTH183 (Mean ± SD)** |  | **Mean ± SD** | **Range** |
| **2016ISG_F** | 0.46 ± 0.02 ^***^ | 0.51 ± 0.02 |  | 0.50 ± 0.03 | 0.35−0.57 |
| **2017ISG_F** | 0.56 ± 0.01 ^*^ | 0.59 ± 0.02 |  | 0.59 ± 0.03 | 0.44−0.65 |
| **2018ISG_F** | 0.57 ± 0.03 ^***^ | 0.62 ± 0.02 |  | 0.60 ± 0.03 | 0.43−0.66 |
| **2017TKB** | 0.43 ± 0.01 ^***^ | 0.52 ± 0.02 |  | 0.51 ± 0.04 | 0.32−0.57 |
| ^***^ and ^*^ represent a significant difference between the two parents at the *P* < 0.001 and 0.05 level based on Student's *t*-test, respectively. | | | | | |

Supplemental Table 3

| **Supplemental Table 3** |  |  |
| --- | --- | --- |
| Average daily temperature during the maturing stage | | |
| Cropping season | IR 64 | YTH183 |
|  | Mean ± S.D. ^1)^ | Mean ± S.D. ^1)^ |
| 2016ISG_F | 29.27 ± 1.01 ^a^ | 29.19 ± 0.97 ^a^ |
| 2017ISG_F | 28.16 ± 1.08 ^a^ | 28.07 ± 1.13 ^a^ |
| 2018ISG_F | 28.02 ± 0.99 ^a^ | 28.02 ± 0.98 ^a^ |
| 2018ISG_S | 24.02 ± 1.21 ^b^ | 23.96 ± 1.15 ^b^ |
| 2017TKB | 23.95 ± 2.38 ^b^ | 24.26 ± 2.63 ^b^ |
| ^1)^ Values followed by the same letter do not differ significantly (*P* < 0.05) among cropping seasons by the Kruskal-Wallis multiple comparison test with Bonferroni correction. | | |
